# 6-week time-restricted eating improves body composition, maintains exercise performance, without exacerbating eating disorder in female DanceSport dancers

**DOI:** 10.1080/15502783.2024.2369613

**Published:** 2024-06-21

**Authors:** Yanbing Zhou, Xian Guo, Zeyao Liu, Dan Sun, Yujie Liang, Hong Shen, Xinxin Li, Jinhao Mu, Jingying Liu, Guoxia Cao, Mengmeng Chen

**Affiliations:** a Beijing Sport University, School of Art, Beijing, China; b Beijing Sport University, Sport Science School, Beijing, China; c Beijing Sport University, Beijing Sports Nutrition Engineering Research Center, Beijing, China; d Beijing Municipal Bureau of Sports, Beijing Lucheng Sports Technical School, Beijing, China; e Beijing Sport University, Competitive Sport School, Beijing, China

**Keywords:** Time-restricted eating, intermittent fasting, exercise performance, fat mass, female dancer

## Abstract

**Background:**

Despite the high risk of eating disorder (ED)-related attitudes and behaviors among female dancers, targeted scientific dietary regimens are currently inadequate. Time-restricted eating (TRE), a popular intermittent fasting protocol, has been shown to be effective in enhancing body composition and exercise performance in athletes. In this study, TRE was employed as a dietary regimen to improve body composition and exercise performance and address ED attitudes and behaviors in DanceSport dancers.

**Methods:**

Twenty female DanceSport dancers were recruited and divided into two groups: TRE (*n* = 10) and normal diet (ND) (*n* = 10). The TRE group consumed their self-selected necessary energy intake exclusively between 11 a.m. and 7 p.m. (utilizing a 16-hour fasting and 8-hour eating window) for 6 weeks, while the ND group maintained their regular dieting patterns. The consumption of water, black tea, or coffee without added sugar or milk was not restricted. Physical activity and calorie intake were systematically recorded during the TRE intervention. Body composition, aerobic and anaerobic performance, and ED attitudes and behaviors were assessed before and after the TRE intervention. The trial was registered in the Chinese Clinical Trial Registry under the identifier ChiCTR2200063780.

**Results:**

The fixed effects tests (*p* < 0.0001) and estimates for the intercept (*p* < 0.0001) of hunger level indicated a noticeable effect on the initial state of hunger during TRE. No significant differences were observed in ED attitudes or behaviors (*p* > 0.05). TRE resulted in a reduction in hip circumference (*p* = 0.039), fat mass (kg) (*p* = 0.0004), and body fat percentage (*p* = 0.0005), with no significant decrease in fat-free mass (*p* > 0.05). No significant improvement was observed in aerobic performance (*p* > 0.05). The average power (AP) (*p* = 0.01) and AP/Body weight ratio (*p* = 0.003) significantly increased. Additionally, the power drop decreased significantly (*p* = 0.019). Group-by-time interactions were observed for fat mass (kg) (*p* = 0.01), body fat percentage (*p* = 0.035), and AP/Body weight (*p* = 0.020).

**Conclusion:**

TRE can be considered a feasible nutritional strategy for DanceSport dancers, facilitating improvements in body composition without compromising aerobic and anaerobic exercise performance or exacerbating ED attitudes and behaviors. Moreover, TRE may facilitate more favorable physiological adaptations, potentially contributing to improved exercise performance.

## 1. Introduction

A judicious nutritional strategy is imperative for dancers [1] because nutrient availability profoundly influences energy expenditure, body composition, and exercise performance [2]. DanceSport is a high-intensity, competitive, and aesthetic form of exercise characterized by strict demands on body shape and exercise performance [3]. The quality and timing of dietary intake are critical for meeting the demands of high-intensity exercise performance [1]. However, dancers often pursue a slim body shape at the expense of physical and mental health and nutrient availability. Numerous studies have demonstrated heightened concern for body shape, eating disorders (ED), and binge-eating behavior among dancers, which are also associated with an increased risk of obesity, heart disease, and diabetes [4,5]. Many female dancers also develop menstrual cycle disorders, osteoporosis, and depression because of their unscientific eating habits [6].

The 16/8 time-restricted eating (TRE) is a specific form of intermittent fasting that limits calorie intake to an 8-hour window with 16 fasting hours per day [7]. TRE allows for an adjustable eating and fasting window, leading to a generally high adherence of up to 87% in human participants [8]. Consequently, TRE has been widely implemented in recent years. Some studies did not find any significant improvements in body composition or exercise performance [9,10]. Numerous studies in athletic groups have suggested that TRE may reduce fat mass (FM) without impairing aerobic and anaerobic performance or fat-free mass (FFM) [11–13]. Some studies have reported neutral results for TRE, without significant improvements in body composition and/or exercise capacity [14–16].

To the best of our knowledge, no previous study has reported the effects of TRE in groups of dancers. Therefore, the aim of our research was to investigate the effects of a 6-week TRE program on female DanceSport dancers. We hypothesized that the TRE protocol would lead to a reduction in FM without impairing aerobic and anaerobic performance or exacerbating eating disorders.

## 2. Methods

### 2.1. Participants

All the participants in this trial were undergraduate DanceSport dancers recruited from the School of Art at Beijing Sport University. They possessed advanced professional techniques, had at least 5 years of training experience, and had participated in domestic or global competitions at least once. They were enrolled in a comparable school curriculum and, as a result, had similar levels of physical activity. The included participants were under 25 years of age, had a body mass index (BMI) of less than 24 kg/m^2^, were not currently using any medication or anabolic steroids, had no acute or chronic diseases, were not undergoing any physical treatment or rehabilitation, and did not smoke or consume alcohol.

All the participants received written information detailing the procedures, requirements, and risks associated with the tests and interventions, which were approved by the Sports Science Experiment Ethics Committee of Beijing Sports University (2022156 H). All participants provided written informed consent before participation.

### 2.2. Study design

**Figure 1.**
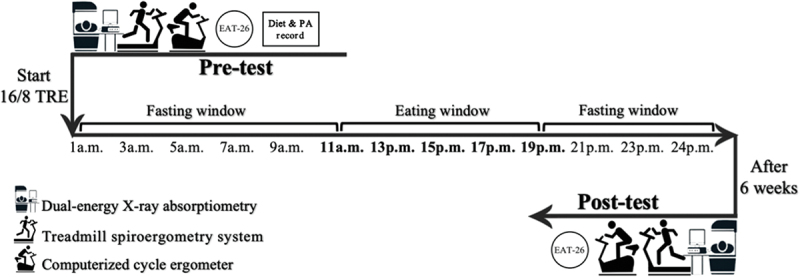
Study design. TRE, Time-restricted eating; PA, Physical activity; EAT-26: Eating attitudes test.

This trial was registered in the Chinese Clinical Trial Registry (https://www.chictr.org.cn.) under the registration number ChiCTR2200063780. The study design is illustrated in Figure 1. The trial comprised a total of 6 nonconsecutive visits, with 3 conducted before the TRE intervention and 3 after the intervention. All measurements were distributed across 3 nonconsecutive visits conducted before and at the conclusion of the 6-week intervention. During the first visit, basic information, height, body weight (BW), and body composition were recorded. Aerobic and anaerobic tests were conducted during the other two visits, with a 5-day interval between visits. All participants commenced the TRE intervention in September 2022.

### 2.3. Sample size

By employing G-Power software (G-Power 3.1.9.6, Developed by Axel Buchner, Heinrich-Heine-Universität Düsseldorf, Germany), the statistical power (1-β) was set at 0.80, the type I error rate (α) at 0.05, the correlation between pre-and-post intervention measures at 0.80, and the effect size at 0.4. The analysis required a sample size of 20 participants (including potential dropouts).

### 2.4. Physical activity intervention

Participants were classified based on their physical activity levels according to their habitual curriculum training routine, which was consistently followed throughout the intervention period. Physical activity levels before and during the TRE intervention were assessed using the short version of the International Physical Activity Questionnaire (IPAQ) [17]. The IPAQ has been validated with reasonable measurement properties for monitoring population levels of physical activity among adults aged 18–65 years in diverse settings [17,18]. The IPAQ was distributed every other week to all participants, and the total weekly metabolic equivalent (MET) was calculated based on the official scoring criteria.

### 2.5. Dietary intervention

All recruited participants were instructed to document all food and liquid intake in pictures and send them via the WeChat application (the most widely used communication application in mainland China) for one week before the TRE intervention, ensuring they maintained normal dietary habits. Participants in the normal diet (ND) group continued with their habitual eating patterns throughout the intervention, usually before their first class at 8 a.m. and after their last class at 8 p.m. Participants in the TRE group were instructed to consume all their dietary intake within an 8-hour window (11 a.m. to 7 p.m.). No energy intake beyond the 8-hour eating window was allowed. The consumption of water, black tea, or coffee without added sugar or milk was not restricted. Throughout the 6-week TRE intervention, all participants were required to document their food and liquid intake in the same manner as before the TRE period, ensuring that the TRE group adhered to the 8-hour window and the ND group maintained their habitual eating patterns. Participants were instructed to send their pictures directly to one researcher rather than in a group chat to minimize interactive influence.

A three-day estimated food record and a 72-hour recall were employed to track and calculate the energy intake in both the TRE and ND groups [19]. Food energy was analyzed using the “BoHe” application, a professional team that offers diet analysis and photo identification of food calories for millions of Chinese people. All subjects reported their energy intake for three consecutive days, including two weekdays and one weekend day (from Thursday to Saturday), both before and every week during the TRE intervention.

Sunday was designated as an energy supplementation day. The three-day estimated food record was converted into calories to assess whether there was an energy deficit between the pretest and the rest of the week. Participants with an energy deficit were instructed to supplement their lack of energy every Sunday by choosing foods containing suitable calories. A dietitian conducted one-on-one consultations with the participants twice a week using the current dietary data to ensure that the progression of food intake in both groups was scientifically managed and did not induce adverse reactions.

### 2.6. Hunger

Throughout the TRE intervention, the hunger levels of participants in the TRE group were recorded using a visual analog scale (VAS) [20]. The VAS was completed every week during the intervention on Saturday just before the TRE eating window began (at 10:30 a.m). The participants were instructed to indicate their current level of hunger by marking the box that best described their feelings. The VAS was equidistantly marked with different levels of hunger: (1) very hungry (need to eat), (2) hungry (would like to eat), (3) somewhat hungry (could eat), (4) no feeling of fullness or hunger, (5) somewhat full (could eat some more), (6) full (I have eaten enough), and (7) very full (feel I have eaten too much).

### 2.7. ED attitudes and behaviors

Eating disorder (ED) attitudes and behaviors were assessed using the Eating Attitudes Test (EAT-26) [21]. The EAT-26 questionnaire comprises 26 statements and 5 behavioral questions. A total score of ≥ 20 in the 26 statements or any endorsement in the 5 behavioral questions would suggest a high risk of developing an ED.

### 2.8. Anthropometry

Height was measured using a wall-mounted stadiometer to the nearest 0.1 cm while subjects stood upright on their heels with shoulders touching the stadiometer. Waist and hip circumferences were measured using heavy-duty inelastic plastic fiber tape, with participants standing with a bare midriff after exhaling, both feet touching, and arms hanging freely [22]. Hip circumference was measured at the maximum extension of the buttocks, and waist circumference was measured directly on the skin at the level of the iliac crest [23]. BW was recorded, and body composition was assessed using a dual-energy X-ray absorptiometry (DXA) scan (Lunar iDXA^TM^, GE Healthcare software enCORE version 16, Madison, WI, USA). The BMI, body fat percentage, FM, and FFM values were recorded. All participants underwent the DXA scan on the same day during both pre- and posttests between 7 and 11 a.m. Participants were instructed to fast for 6 hours and abstain from consuming any liquid for 2 hours prior to the testing session. After changing into light clothes and removing shoes and accessories, the participants were instructed to lie in a supine position with their feet evenly positioned on either side of the midline and arms placed equidistant to the trunk with hands pronated and fingers spread. Before the tests, a quality assurance program was implemented with known properties, in accordance with the manufacturer’s instructions.

### 2.9. Aerobic performance

The incremental exercise test was conducted on a motorized treadmill (RUN 7410/T-PC; Runner^TM^, Cavezzo MO, Italy). Following a comprehensive oral explanation of the test, sufficient stretching preparation, and a 3-minute warm-up at 7 km/h, the speed was increased by 1 km/h after every 3-minute interval. This protocol provided the most accurate submaximal measurement of physiological variables [24], until volitional exhaustion [25]. Ratings of perceived exertion (RPE) and heart rate (HR) were recorded during the last 30 seconds of each stage. A 1% gradient was maintained throughout the test to simulate outdoor running [26]. The test was conducted in a scientific motor laboratory with a room temperature between 21–24 degree Celsius. Respiratory gas exchange parameters including maximal oxygen intake (VO_2max_), oxygen intake per kilogram (VO_2_/kg), carbon dioxide production (VCO_2_), and anaerobic threshold (AT) were recorded. The VO_2max_ was determined if any of the two following criteria were met: a respiratory exchange ratio over 1.1; an HR ±10 bpm of the maximal age-predicted HR; RPE scale over 18; oxygen intake of the subjects increased to the plateau period or slowly decreased with the load intensity [27].

### 2.10. Anaerobic performance

The Wingate Anaerobic Test was conducted using a computerized cycle ergometer (Monark 894E Peak Bike, Monark TM, Sweden). Matching toe clips are installed to prevent foot slippage. The foot position on the pedals, saddle height, and upper-body posture were customized according to the individual anthropometric specifications. The individual adjustments remained the same during both pre- and posttests. Following an oral explanation of the test and a 10-minute warm-up, the participants were instructed to pedal at full speed with the cycle ergometer unloaded for 5 seconds. During this stage, a braking force equivalent to 7.5% of individual’s body mass was applied with a countdown of 30 seconds [28]. Throughout the test, the participants were verbally encouraged to achieve their best performance. The peak power (PP), PP/BW, average power (AP), AP/BW, and power drop (PD) were recorded. PD was calculated using this equation: Power Drop (%) = ((Peak Power − Minimum Power)/Peak Power) × 100, where PP and minimum power represent the highest and lowest power output achieved during the Wingate test [29]. The test was conducted in a scientific motor laboratory with a room temperature between 21–24 degrees Celsius.

### 2.11. Statistics

Statistical analysis was conducted using SPSS software (IBM SPSS Statistics 27.0, Ehningen, Germany). Results were presented as means ± SD or n (%). Normal distribution was assessed using Shapiro-Wilk’s W test. An independent samples t-test was used to assess baseline differences between the groups. A repeated-measures ANOVA with group-by-time interaction was used to assess the intervention effects. Outliers and sphericity were assessed before conducting repeated-measures ANOVA. A Chi-square test (χ^2^) was utilized for non-parametric analysis. A linear mixed-effects model was applied, considering time as a fixed effect to capture the overall trend across multiple time points, while accounting for individual variability through random effects. Differences were considered statistically significant at *p* < 0.05.

## 3. Results

### 3.1. Characteristics and physical activity

A total of 20 female DanceSport dancers (age: 19.25 ± 1.21 years, *p* = 0.37; height: 1.65 ± 0.04 cm, *p* = 0.51; body mass: 52.52 ± 5.28 kg, *p* = 0.30; body mass index: 19.21 ± 1.75 kg/m^2^, *p* = 0.44) enrolled at Beijing Sport University were recruited and randomly assigned to the TRE (*n* = 10) or ND (*n* = 10) group using computer-generated software. No dropouts were recorded, and all participants completed the TRE intervention and the pre- and posttests. No reports of side effects were received or detected by the researcher or dietitian during or after the intervention. The baseline characteristics of each group are shown in Table 1. No significant differences were observed at the baseline.

**Table 1.** Baseline characteristics.

	TRE (*n* = 10)	ND (*n* = 10)
Age (years)	19.0 ± 0.9	19.5 ± 1.4
Height (m)	165.9 ± 4.2	164.8 ± 3.1
Body weight (kg)	53.8 ± 6.2	51.3 ± 4.1
BMI (kg/m^2^)	19.5 ± 2.0	18.9 ± 1.6
Physical activity (MET-min/week)	10413.5 ± 3807.6	9143.1 ± 4814.4

The participants spent 3.2 ± 1.4 hours per day on DanceSport training and had a baseline physical activity level of 9778.3 ± 4274.5 MET-min/week. During the TRE intervention, the TRE group performed 10,003.1 ± 3710.1 MET-min/week of physical activity, while the ND group engaged in 8953.9 ± 4657 MET-min/week. No significant differences were observed (*p* = 0.521).

### 3.2. Hunger

**Figure 2.**
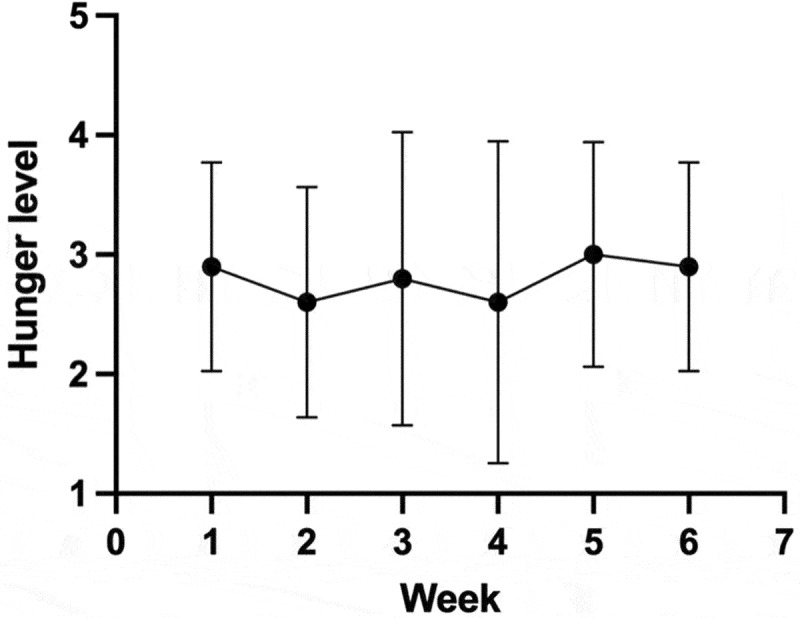
The line chart of hunger levels in TRE group during the 6-week TRE intervention.

Hunger levels during the 6-week TRE intervention were recorded, as shown in Figure 2. According to the linear mixed-effects model, the covariance parameter estimates for residual variance were highly significant (Z = 4.415, *p* < 0.0001), indicating a good fit for individual variability. The fixed-effects tests demonstrated a highly significant intercept (F = 75.669, *p* < 0.0001) and the fixed-effect estimates for the intercept were also highly significant (*t* = 8.699, *p* < 0.0001). The fixed-effect estimate for the different testing sessions (time effect) was not statistically significant (*t* = 0.473, *p* = 0.643).

### 3.3. Energy intake

The three-day estimated food records and 72-hour recalls before and during TRE intervention were analyzed. As shown in Table 2, no significant differences were found in the total calorie intake and macronutrients between the groups. Moreover, no significant differences in total calorie and macronutrients intake were observed between the baseline level and during the TRE intervention (total calorie intake at baseline: TRE: 1486.4 ± 215.6 kcal; ND: 1562.2 ± 323.8 kcal). During the TRE intervention, the TRE group consumed an average of 1523.6 ± 333.1 kcal per day. Of these, 15.2 ± 2.2% was protein, 32.8 ± 3.4% was fat, and 52.0 ± 4.2% was carbohydrates. The ND group ingested 1595.9 ± 328.2 kcal per day during the experiment, with 16.7 ± 2.1% from protein, 34.2 ± 3.2% from fat, and 49.1 ± 4.4% from carbohydrate.

**Table 2.** Total calorie intake and macronutrients at baseline and during TRE in TRE and ND group.

	Baseline		During TRE	
	TRE (*n* = 10)	ND (*n* = 10)	TRE (*n* = 10)	ND (*n* = 10)
Total calories (kcal/day)	1486.4 ± 215.6	1562.2 ± 323.8	1523.6 ± 333.1	1595.9 ± 328.2
Protein (kcal/day)	234.9 ± 10.9	229.6 ± 10.0	231.6 ± 7.3	266.5 ± 6.9
Protein (%)	15.8 ± 5.1	14.7 ± 3.1	15.2 ± 2.2	16.7 ± 2.1
Fat (kcal/day)	475.7 ± 14.2	539.0 ± 33.7	500.0 ± 11.3	545.8 ± 10.5
Fat (%)	32.0 ± 6.6	34.5 ± 10.4	32.8 ± 3.4	34.2 ± 3.2
Carbohydrate (kcal/day)	775.9 ± 17.9	792.0 ± 33.0	792.3 ± 14.0	783.6 ± 14.4
Carbohydrate (%)	52.2 ± 8.3	50.7 ± 10.2	52.0 ± 4.2	49.1 ± 4.4

### 3.4. ED attitudes and behaviors

ED attitudes and behaviors at baseline and after 6 weeks in the TRE and ND groups are shown in Table 3. The overall ED level, binge eating behavior, and significant weight loss behavior (20 pounds or more in the last 6 months) remained the same at baseline and after 6 weeks. No significant differences were observed between baseline and posttest in vomiting behavior (*p* = 0.5), drug use behavior (*p* = 1.582), or excessive exercise behavior (*p* = 0.474).

**Table 3.** ED attitudes and behaviors at baseline and posttest in TRE and ND group.

	Baseline			Posttest		
	TRE (*n* = 10)	ND (*n* = 10)	χ^2^	TRE (*n* = 10)	ND (*n* = 10)	χ^2^
A high level of concern about dieting, body weight or problematic eating behaviors						
Endorsed	2 (20%)	3 (30%)	0.267	2 (20%)	3 (30%)	0.267
Not Endorsed	8 (80%)	7 (70%)		8 (80%)	7 (70%)	
Gone on eating binges where you feel that you may not be able to stop						
Endorsed	3 (30%)	4 (40%)	0.220	4 (40%)	2 (20%)	0.952
Not Endorsed	7 (70%)	6 (60%)		6 (60%)	8 (80%)	
Ever made yourself sick (vomited) to control your weight or shape						
Endorsed	1 (10%)	2 (20%)	0.392	3 (30%)	1 (10%)	1.250
Not Endorsed	9 (90%)	8 (80%)		7 (70%)	9 (90%)	
Ever used laxatives, diet pills, or diuretics to control your weight or shape						
Endorsed	3 (30%)	1 (10%)	1.250	3 (30%)	2 (20%)	0.267
Not Endorsed	7 (70%)	9 (90%)		7 (70%)	8 (80%)	
Exercised more than 60 minutes a day to lose or to control your weight						
Endorsed	2 (20%)	0 (0%)	2.222	0 (0%)	1 (10%)	1.053
Not Endorsed	8 (80%)	10 (100%)		10 (100%)	9 (90%)	
Lost 20 pounds or more in past 6 months						
Endorsed	1 (10%)	1 (10%)	0.200	1 (10%)	1 (10%)	0.200
Not Endorsed	9 (90%)	9 (90%)		9 (90%)	9 (90%)	

### 3.5. Body composition

**Figure 3.**
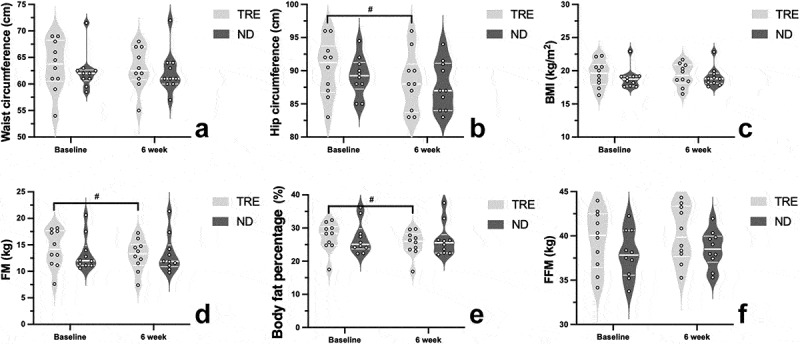
The violin plots of changes of waist circumference, hip circumference, body mass index (BMI), fat mass (FM) and fat-free mass (FFM) in TRE and ND group at baseline and after TRE intervention. The thick lines indicate the median value in each group. The thin lines represent each interquartile range. The pound sign (#) illustrates within-group differences (*p* < 0.05).

The body composition at baseline and after 6 weeks in the TRE and ND groups is shown in Figure 3. No significant differences were found in waist circumference (*p* = 0.70) (Figure 3a), BMI (*p* = 0.72) (Figure 3c), or FFM (*p* = 0.88) (Figure 3f). The hip circumference (*p* = 0.039) (Figure 3b) differed significantly after 6 weeks, changing from 90.4 ± 4.3 cm to 88.3 ± 4.4 cm. Meanwhile, FM (kg) (*p* = 0.0004) (Figure 3d) and body fat percentage (*p* = 0.0005) (Figure 3e) were significantly altered after 6 weeks, changing from 14.3 ± 3.6 kg and 27.3 ± 4.4% to 13.0 ± 2.9 kg and 25.4 ± 3.8%, respectively. In the repeated measures ANOVA, significant group-by-time interactions were observed for FM (kg) (F = 8.371, *p* = 0.01) and body fat percentage (F = 5.172, *p* = 0.035). No other significant group-by-time interactions were observed for body composition assessments.

### 3.6. Aerobic performance

**Figure 4.**
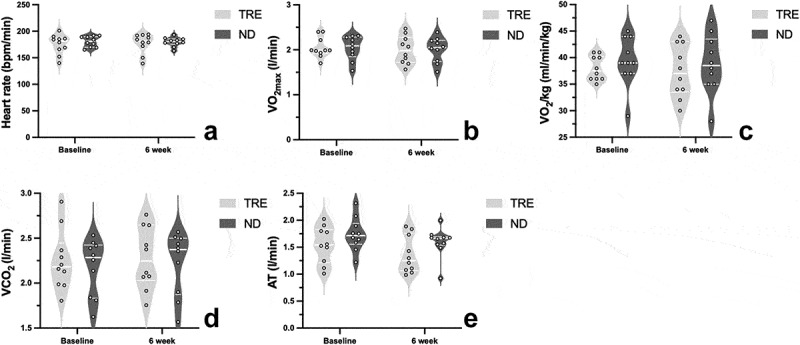
The violin plots of changes of heart rate (HR), maximum oxygen intake (VO_2max_), oxygen intake (VO_2_/kg), carbon dioxide production (VCO_2_) and anaerobic threshold (AT) in TRE and ND group at baseline and after TRE intervention. The thick lines indicate the median value in each group. The thin lines represent each interquartile range.

Figure 4 shows the aerobic performance of the TRE and ND groups at baseline and after 6 weeks. No significant between-group or within-group differences were observed in HR (*p* = 0.68) (Figure 4a), VO_2max_ (*p* = 0.97) (Figure 4b), VO_2_/kg (*p* = 0.56) (Figure 4c), VCO_2_ (*p* = 0.73) (Figure 4d), or AT (*p* = 0.11) (Figure 4e). In the repeated-measures ANOVA, no significant group-by-time interaction was observed in aerobic performance assessments.

### 3.7. Anaerobic performance

**Figure 5.**
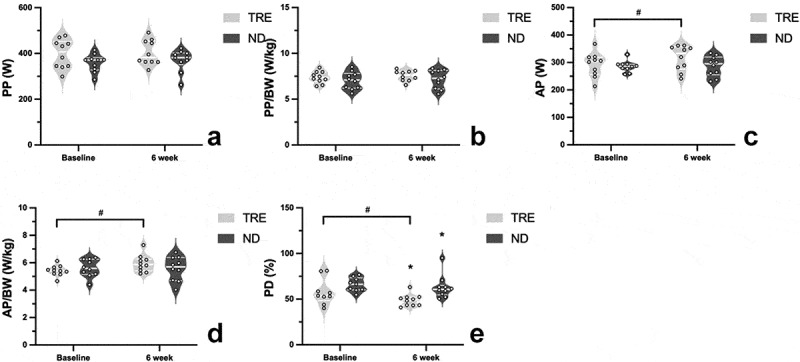
The violin plots of changes of peak power (PP), PP/BW, average power (AP), AP/BW and power drop (PD) in TRE and ND group at baseline and after TRE intervention. The thick lines indicate the median value in each group. The thin lines represent each interquartile range. The asterisk mark (*) demonstrates between-group significant difference (*p* < 0.05). The pound sign (#) illustrates within-group differences (*p* < 0.05).

Anaerobic performance at baseline and after 6 weeks in the TRE and ND groups is shown in Figure 5. Baseline data from one subject each in the TRE and ND groups were excluded because they were verified as outliers. No significant differences were found in PP (*p* = 0.17) (Figure 5a) or PP/BW (*p* = 0.30) (Figure 5b). The within-group significant differences in AP (*p* = 0.01) (Figure 5c) and AP/BW (*p* = 0.003) (Figure 5d) were observed, changed from 292.1 ± 43.9 W to 316.5 ± 45.8 W and from 5.4 ± 0.4 W/kg to 5.9 ± 0.6 W/kg separately. The within-group PD after 6 weeks was significantly differed (TRE: 57.9 ± 14.3%; ND: 66.0 ± 7.48%) (*p* = 0.043) (Figure 3e). Moreover, significant decrease of PD was also detected in TRE group after 6 weeks compared with ND group (*p* = 0.05), altered from 62.6 ± 20.1% to 48.8 ± 6.5% (Figure 3e). In the repeated measures ANOVA, significant group-by-time interactions were observed for AP/BW (F = 6.529, *p* = 0.020). No other significant group-by-time interactions were observed during anaerobic performance assessment.

## 4. Discussion

To the best of our knowledge, this is the first TRE intervention study specifically targeting DanceSport dancers. Dancer groups, particularly female dancers, face high demands regarding body shape and exercise performance. Consequently, the daily intake of fats and carbohydrates may be more frequently restricted than in other groups [30]. Recent research reported an approximately 50% underweight rate among female ballet dancers and artistic gymnasts [30], leading to a higher risk of disordered eating attitudes and low energy availability [30–32]. Our study demonstrated that a 6-week 16/8 TRE intervention reduced FM while maintaining FFM, without impairing aerobic or anaerobic performance. AP/BW increased and PD decreased after the 6-week intervention period. Additionally, no significant improvement was observed in ED attitudes or behaviors.

Hunger levels in the TRE group were recorded weekly during the 6-week intervention. The highly significant intercept for both the fixed effects analysis and estimation, combined with the non-significant results for the time effect, suggests that engaging in TRE had a remarkable effect on the initial state of hunger. However, hunger levels did not exhibit a significant trend over the six-week period, which means that the hunger impact of TRE may be more pronounced at the beginning of the intervention but remains relatively stable over time, reducing the improvement in adherence and making TRE more subjectively acceptable.

Consistent with most previous studies, in this study, FM in kilograms and as a percentage significantly decreased after TRE, with no impairment in FFM. In addition, a significant decrease in hip circumference was observed in the TRE group. Although not every TRE intervention results in significant changes in body composition [14], various durations of TRE ranging from 4 to 8 weeks have been shown to significantly reduce BW or FM without affecting exercise performance capacity [12,14,33]. Notably, unlike interventions involving calorie restrictions, for example, a 25% caloric deficit [14] or instructing participants to consume isocaloric diets with the same macronutrient composition [33], our study did not restrict calorie intake during the entire TRE intervention. Participants were allowed to consume as much self-picked food as desired within an 8-hour eating window. Hatori et al. [34] found an increase in hepatic AMP and phospho-acetyl coenzyme A carboxylase (pACC), reflecting increased hepatic AMPK activity in mice fed a high-fat diet with 16/8 TRE (no change in energy intervention). This may be the mechanism by which TRE regulates metabolism without altering the energy intake.

TRE significantly reduced FM in this study, potentially because of its impact on circadian rhythms, thereby influencing energy expenditure rates [35,36]. When combined with exercise, TRE has the potential to enhance lipid metabolism, thereby contributing to alterations in glucose and fat consumption rates [37,38]. Although the main increase in lipid metabolism occurs between 18 and 24 h of acute fasting [39], significant changes can occur during long-term intervention with shorter fasting durations. By coordinating feeding times and leveraging circadian rhythm, TRE activates key metabolic regulators and pathways involved in lipid metabolism, such as PPAR γ and AMPK. For example, TRE increases the level of REV-ERB α (a circadian transcriptional repressor) and reduces expression of a key lipogenic gene, fatty acid synthase [40]. The function of the AMPK pathway, 5′-cyclic adenosine 5′-monophosphate response element binding protein, and mammalian target of rapamycin, as well as the circadian clock oscillations and target gene expression, can all be enhanced by TRE. Liver metabolism is modified, and food uptake and energy expenditure are enhanced by these modifications to the anabolic and catabolic pathways [34].

Despite the relatively short duration of fasting (6 weeks), time-restricted eating (TRE) can positively impact body composition and exercise performance through several mechanisms [39]. TRE enhances fat oxidation, leading to a reduction in body fat mass, while also improving insulin sensitivity. This enhancement facilitates glucose uptake by muscle cells and aids in exercise performance and recovery. Additionally, an increase in growth hormone secretion during fasting can promote muscle growth and repair, potentially enhancing exercise performance. Furthermore, the reduction in inflammation and improvement in mitochondrial function resulting from TRE can contribute to a better recovery from exercise-induced muscle damage and enhance energy production during physical activity [41].

DanceSport, as a sports discipline, involves alternating physical activity with medium duration and high energy demands, encompassing both aerobic and anaerobic phases separated by short recovery periods [25]. The current study did not observe any significant improvements in aerobic capacity. While practical evidence in dancers is still limited, similar supportive results were achieved in an 8-week TRE experiment in runners; exercise performance was not impaired with a decrease in body weight and energy intake [12]. They observed no improvement in laboratory-based measures of aerobic performance. As reported, 4–8 weeks of TRE intervention in endurance or resistance training athletes did not result in any significant improvement in running economy or •VO_2max_ values during a graded maximal exercise test [11–13]. Consistent with a previous study, adherence to a TRE diet had a neutral effect on aerobic performance [33]. In our study, improvements in aerobic performance were more closely related to exercise training and less affected by short-term dietary interventions. Although the participants in this study also underwent normal training, the 6-week TRE intervention had little effect on their aerobic performance.

In our study, anaerobic AP and AP/BW ratio significantly improved with a decrease in PD. Similar results were also found in a 4-week TRE intervention in healthy men; the Wingate tests were conducted after 1 and 4 weeks of TRE intervention, and a significant increase in AP was only observed after 4 weeks [42]. This suggests that short-term TRE can lead to the depletion of hepatic glycogen, which may negatively impact exercise performance [9]. Furthermore, TRE can elicit better physiological adaptations to enhance anaerobic exercise performance, and this effect is strongly mediated by TRE [42]. This finding was also supported by Park et al. [43]. The higher glycogen content in the liver and skeletal muscles resulting from intermittent fasting may be the reason for the improvement in AP and AP/BW in the present study.

PD is defined as the difference between PP and minimum power, and originally demonstrated physical fatigue during exercise [44]. Numerous studies have shown that because of the metabolic switch that occurs during a change in diet regimen, TRE could lead to decreased weight gain and lower blood glucose levels [45]. This, in turn, increases beta-hydroxybutyrate levels during lipid metabolism, resulting in higher ketone levels and, consequently, resistance to physical fatigue [46,47].

This study was designed for DanceSport dancers with the original aim of improving their physical and mental health, which is often exacerbated by unhealthy eating habits, such as restricted food intake or binge eating behavior, owing to their high requirement for body shape and weight. Despite numerous studies proving the efficacy of TRE in athletic groups or exercise performance [10,12,13,33,42,48], a clear prevalence of ED among female dancers has been reported in previous studies [49,50]. Therefore, the EAT-26 was administered in this study before and after the 6-week TRE intervention to address psychological safety and ensure that this eating strategy would not mask any risky behaviors. Based on these results, TRE, despite being an eating restriction regimen for DanceSport dancers, a high-risk group, could be considered a feasible and effective dietary strategy to reduce their unhealthy eating habits.

## 5. Limitations

This study had some limitations. (1) As a pilot study with 20 participants, the results can be justified by further research with larger samples, whether through randomized controlled trials or cohort studies. (2) The energy and macronutrient intake in this study were estimated using a consecutive three-day estimated food record and a 72-hour recall, which may have limitations in assessing the accuracy of nutritional calculations. (3) Since blinding of the participants was not possible with a TRE intervention and all the included subjects were undergraduate DanceSport dancers who might know each other in the same grade, potential interactions between groups might have existed, which could have influenced how they performed the intervention. Although the participants were coded with numbers, researchers were not blinded to the group participants when performing the exercise performance tests, potentially introducing bias. (4) despite no exacerbation of ED was observed, other potential psychological effects cannot be ruled out and should be further analyzed in future studies. (5) A recent study reported that the improvement in total body mass, insulin resistance, fasting glucose, adiposity, inflammation, and gut microbial diversity was better in the early TRE (food intake restricted to the early part of the day) than in the middle TRE (food intake restricted to the middle of the day) [51]. We selected the middle TRE in this study to align with the curriculum of dancers in schools. Further studies are needed to investigate the effects of early TRE and compare different TRE regimens.

## 6. Conclusions

In conclusion, our study suggests that the 16/8 TRE protocol is a feasible nutritional regimen for DanceSport dancers. This approach has the potential to enhance body composition while concurrently sustaining aerobic and anaerobic exercise performance without exacerbating eating disorders.
